# Biallelic pathogenic hydroxymethylbilane synthase gene variants of a neurodegenerative disorder with progressive cystic leukoencephalopathy: a case report

**DOI:** 10.1186/s13256-026-05879-2

**Published:** 2026-02-23

**Authors:** Gabriel Schacht, Miriam Elbracht, Anna-Elisabeth Minder, Thomas Stauch, Arzu Stoppe, Eva Lausberg, Martin Häusler

**Affiliations:** 1 Department of Pediatrics, Division of Neuropediatrics and Social Pediatrics, University Hospital RWTH Aachen, Aachen, Germany; 2 Institute for Human Genetics and Genomic Medicine, Medical Faculty, RWTH Aachen University, Aachen, Germany; 3 Division of Endocrinology, Diabetology, Porphyria, Stadtspital Zurich, Triemli, Zurich, Switzerland; 4 MVZ Labor PD Dr. Volkmann GbR, Karlsruhe, Germany

**Keywords:** Porphyria, Leukoencephalopathy, Hydroxymethylbilane synthase, 5-aminolevulinic acid, Case report

## Abstract

**Background:**

Heterozygous mutations of the hydroxymethylbilane synthase gene can lead to acute intermittent porphyria, with episodic abdominal pain and neuropsychiatric symptoms. The heme precursors 5-aminolevulinic acid and porphobilinogen accumulate due to enzyme deficiency. Case reports of biallelic pathogenic hydroxymethylbilane synthase gene variants are very rare.

**Methods:**

This case report presents a severely affected boy with biallelic pathogenic hydroxymethylbilane synthase gene variants and includes literature overview of other case reports and experimental data.

**Case presentation:**

At the age of 2 years, a Caucasian boy with pathologic psychomotor development was diagnosed with biallelic pathogenic hydroxymethylbilane synthase gene variants. As in previous case reports, he did not exhibit symptoms of acute intermittent porphyria, but progressive cystic leukoencephalopathy and neurological decay. In his urine, 5-aminolevulinic acid and porphobilinogen were markedly elevated, but in cerebrospinal fluid just porphobilinogen.

**Literature review:**

Data of human and animal studies indicate that neurologic symptoms of acute intermittent porphyria are caused by 5-aminolevulinic acid, which episodically accumulates from hepatic origin. Here, as long-term treatment, the inhibition of hepatic heme synthesis with the small interfering RNA givosiran has proven to be effective. In case of biallelic pathogenic hydroxymethylbilane synthase gene variants, the heme precursors 5-aminolevulinic acid or porphobilinogen originating from the liver or central nervous system could be causative, and absolute heme deficiency in the central nervous system is another hypothesis. However, parenterally administered heme, which is effective in acute intermittent porphyria, does not reach the central nervous system. In one case of biallelic pathogenic hydroxymethylbilane synthase gene variants, a liver transplantation did not lead to long-term benefit.

**Conclusion:**

For differential diagnosis of cystic leukoencephalopathy, biallelic pathogenic hydroxymethylbilane synthase gene variants should be considered. Its pathogenesis probably differentiates from acute intermittent porphyria. To date, there is no promising therapeutic approach.

**Supplementary Information:**

The online version contains supplementary material available at 10.1186/s13256-026-05879-2.

## Background

The third enzyme of the heme biosynthesis pathway is hydroxymethylbilane synthase (HMBS, also known as porphobilinogen deaminase), which catalyzes the transformation of porphobilinogen (PBG) to its linear tetramer hydroxymethylbilane and is encoded by the HMBS gene [1]. Heterozygous pathogenic variants of HMBS can lead to acute intermittent porphyria (AIP), characterized by episodic abdominal pain, psychiatric symptoms, and peripheral neuropathy. These attacks are often triggered by dieting, stress, distinct drugs, or xenobiotics. The pathogenesis of these attacks is not completely understood. Acute accumulation of PBG and 5-aminolevulinic acid (ALA), which cannot be adequately metabolized because of HMBS deficiency, is one main hypothesis [2, 3], but a lack of neuronal heme might also be important [4, 5]. The treatment of an acute attack includes heme infusion to induce negative feedback. Givosiran, an ALA synthase 1 (ALAS1) small interfering RNA (siRNA) that inhibits the hepatic heme synthesis, reduces the frequency of porphyric attacks as long-term treatment [6–9].

Referring to a prospective study from Elder *et al*., symptomatic AIP is very rare, with an incidence in Europe of at least 0.13 per million per year (95% CI 0.10–0.14), and a prevalence of at least 5.4 per million (95% CI 4.5–6.3). The study also showed that AIP is more common in northern Sweden [10]. A French study, evaluating the prevalence of nonsymptomatic carriers of pathogenic HMBS variants, estimated a prevalence of 1:1.675, by identifying two unrelated heterozygous carriers out of 3.350 individuals [11], which translates into a pathogenic allele frequency of 0.0006. The analysis of a genomic database of 45.955 Caucasians for HMBS heterozygous carriers revealed a pathogenic allele frequency of 0.00056, resulting in an extremely low clinical penetrance of 1% for AIP, using the prevalence estimated by Elder *et al*. [10, 12]. It is known that environmental factors, such as specific drugs and potentially genetic and epigenetic variants, play a role in the clinical manifestation of the disease [13]. As a genetic susceptibility factor, cytochrome variants were associated with a higher penetrance [14].

Not all details of the catalytic process, enabled by HMBS, have been solved yet. Several studies, however, show that the protein structure of monomeric HMBS consists of three domains, interacting with the cofactor dipyrromethane. Structural changes in any of these domains can lead to diminished enzyme activity and AIP [15, 16]. Most of the variants, which have been reported as causative for AIP, are missense mutations (31.9%, data from 2020), and the remaining variants include small deletions, splicing defects, small insertions, nonsense variants, and others [15].

Thus far, only 13 cases with biallelic pathogenic HMBS variants (B-HMBS-V) have been published [17–25]. Despite showing highly elevated concentrations of PBG, ALA, and other heme precursors, most of them did not present acute porphyric attacks, but suffered from a neurodegenerative disorder with spastic paraparesis and leukoencephalopathy, and some also from peripheral neuropathy. Bilateral cataracts, optic nerve atrophy, and cerebellar abnormalities were also common. The age at onset varied between a few months and 13 years. Whereas some reached adulthood, others died in their first decade of life [17–25]. Although HMBS is known as a tumor suppressor gene and patients with AIP have an elevated risk for hepatocellular carcinoma (HCC) associated with a second somatic mutation in the tumor tissue [26], HCC has not yet been reported in patients with B-HMBS-V [27–29].

The cause of brain damage in B-HMBS-V is still unclear, and assumptions as to the pathophysiology are similar to those about the genesis of acute porphyric attacks in AIP. One hypothesis attributes the phenotype to heme precursors, which are pathologically elevated due to HMBS deficiency. In line with this hypothesis, *in vivo* and *in vitro* studies revealed a modification of neuronal activity mediated by gamma-aminobutyric acid (GABA) due to ALA [30–32]. However, several studies indicate a low permeability of the blood brain barrier for ALA and PBG [33–35]. Another hypothesis speculates that the lack of neuronal heme may cause neuronal damage [1].

There are models of genetically engineered mice with B-HMBS-V. Several of these models also developed peripheral neuropathy, motor developmental delay, and defective myelination, hence it could be assumed that the murine data are useful for the understanding of the pathophysiology of human B-HMBS-V [35–38].

HMBS is not the only enzyme of heme biosynthesis, whose deficiency may provoke leukoencephalopathy. A degeneration of white matter was also seen in some cases of homozygous variants in the PPOX gene and childhood onset variegate porphyria (VP) [39–43]. VP, characterized by protoporphyrinogen oxidase deficiency, the penultimate enzyme of the heme biosynthesis pathway, also leads to the elevation of PBG and ALA. Common features of patients with homozygous VP are nystagmus, developmental delay, brachydactyly, and reduction of myelin on magnetic resonance imaging (MRI). These patients additionally present with photosensitivity and skin abnormalities, which are the main features of heterozygous VP [43].

Here we present a further severely affected child with B-HMBS-V. Our analysis highlights the potential importance of PBG in the pathophysiology of the neurodegenerative disease, categorizes the genetics of B-HMBS-V, and evaluates current treatment options.

## Methods

### Aim of the study

This study describes the disease, caused by B-HMBS-V, as a literature review and presents a new case of the rare disorder. It aims to elaborate the current knowledge about phenotypic characteristics, pathophysiology, and genotype–phenotype correlation of B-HMBS-V. In this context, possible therapeutic approaches are evaluated.

### Editorial policies and ethical considerations

All procedures were performed in compliance with relevant laws, institutional guidelines, and ethics recommendations. The privacy rights of human subjects have always been observed, and informed consent of the patient’s parents was obtained. The work described has been carried out in accordance with the Code of Ethics of the World Medical Association (Declaration of Helsinki) for experiments involving humans. The study was approved by the ethics committee of the medical faculty, RWTH Aachen (reference no. EK 24–066).

### Biochemical studies

Determinations of porphyrin precursors (ALA and PBG) were carried out using a commercially available test kit (ClinEasy®, Recipe Chemicals + Instruments GmbH, Munich) according to the method of Mauzerall and Granick [44]. Porphyrin analysis was done by high-performance liquid chromatography (HPLC) with fluorescence detection on a Waters Alliance system following a procedure of Armbruster *et al.* [45]. The activity of HMBS in blood was measured according to Doss and Tiepermann by photometric determination of uroporphyrin after heat inactivation of uroporphyrinogen decarboxylase in a hemolyzed erythrocyte pellet [46].

### Genetic studies

Next-generation sequencing analysis was performed using short-read sequencing technology (Illumina) to detect DNA alterations in coding regions and adjacent intron–exon segments. A probe-based capture method was used to enrich the target regions (exome, intron–exon-boundaries) (IDT, xGen Exome Research Panel v2.0). Analysis of both single base variants and copy number variants was performed using an in-house pipeline on the basis of SeqMule (http://seqmule.openbioinformatics.org/en/latest) and CNVKit (https://cnvkit.readthedocs.io/en/stable/), respectively, and alignment with the human reference genome (hg38). Variant analysis and prioritization were performed using the Kggseq software (http://grass.cgs.hku.hk/limx/kggseq/) or the SeqPilot module (SeqPatient, SeqNEXT/CNV module, JSI, genome build hg19).

## Case presentation

The patient reported here is a Caucasian boy who was born at term after an uneventful pregnancy. No metabolic or neurological diseases were known in his healthy, unrelated parents, his two older siblings, or further family members. Delayed motor development with marked muscular hypotonia was noted in his first year of life. He started to crawl at the age of 1 year. After 2 years of life, he was able to straighten up and to stand with assistance. During the following 2 years, he lost the ability to stand with assistance due to upcoming spastic tetraparesis. He did not learn to speak. At the age of 1 year and 11 months and at the age of 4 years, he suffered from generalized epileptic seizures. Ophthalmologic studies revealed pale optic discs. For comparison of the patient’s findings with literature data, see Table 1 of the Additional file 1.

Next-generation sequencing and subsequent segregation analysis revealed biallelic pathogenic variants in Exon 10 of HMBS, NM_000275.2: c.499C > T, p.(R167W) (maternal) and c.500G > A, p.(R167Q) (paternal), respectively. Both parents did not exhibit any clinical signs of AIP.

Urine studies for heme metabolites, performed at the age of 2 years and 11 months, revealed markedly elevated concentrations for ALA (240.8 μmol/g creatinine; normal: < 25.0 μmol/g creatinine), PBG (483.3 μmol/g creatinine; normal: < 8.0 μmol/g creatinine), uroporphyrin (957.2 nmol/g creatinine; normal < 38.3 nmol/g creatinine), heptacarboxyporphyrin (28.6 nmol/g creatinine; normal < 12.0 nmol/g creatinine), and pentacarboxyporphyrin (53.3 nmol/g creatinine; normal < 7.2 nmol/g creatinine), whereas coproporphyrin (125.3 nmol/g creatinine; normal < 182.8 nmol/g creatinine) was not elevated. The amount of total porphyrins in urine was elevated to 1191 nmol/g creatinine (normal < 250 nmol/g creatinine). HMBS enzyme activity in the erythrocytes was reduced to 16% (3.1 nmol/l/s; normal 13.3–24.7 nmol/l/s). There were no elevations of liver enzymes or clinical signs of liver dysfunction. Cerebrospinal fluid (CSF) analyses, performed twice, displayed normal values for cells, glucose, and protein, whereas lactic acid was slightly increased at the age of 1 year and 11 months (3.7 mmol/l; normal 1.1–2.8 mmol/l) and normal at the age of 2 years and 10 months (2.6 mmol/l; normal 1.1–2.8 mmol/l). Serum lactic acid, in contrast, proved elevated in three of seven samples tested (2.8, 2.8 and 3.7 mmol/l). At the age of 2 years and 10 months, CSF was also studied for ALA and PBG levels. As no CSF reference values were available, the levels were compared with serum reference values. Hereby both concentrations were increased, but PBG to a much greater extent than ALA, indicating an accumulation of PBG in the CSF compartment (ALA: 0.6 μmol/l, normal in serum 0.36–0.41 μmol/l; PBG: 3.65 μmol/l, normal in serum < 0.12 μmol/l) [47].

**Fig. 1:**
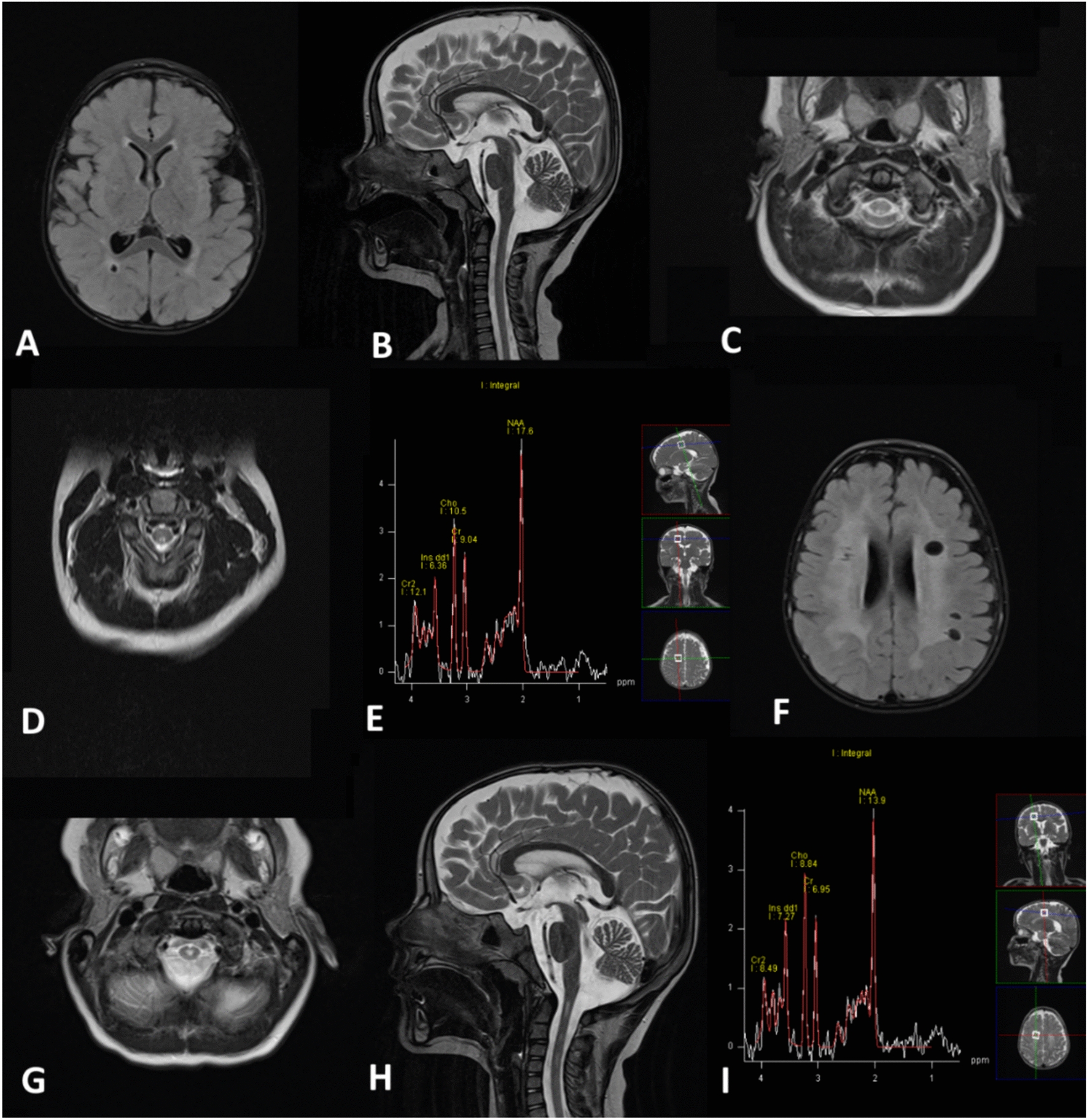
Magnetic resonance imaging. Age 1 year and 11 months: T2 high signal changes in the bilateral white matter with occipital accentuation, right temporo-occipital periventricular cyst (**A**). T2 high signal changes of the bilateral dorsal columns, extending from C1 to C5 (**B**, **C**). Age 2 years and 4 months: supratentorial white matter changes and spinal findings still present (**D**). Increased myo-inositol levels on long echo time magnetic resonance spectroscopy from temporo-parietal white matter (**E**) (Spin Echo 135 ms, 8 ml single voxel study). Age 3 years and 9 months: increase of supratentorial T2 high signal white matter changes, of white matter cysts (**F**), and of spinal T2 high signal changes, now located more centrally (**G**, **H**). Persisting increase of myo-inositol levels on MR spectroscopy (**I**)

At the age of 1 year and 11 months, brain MRI showed T2 high signal changes in the bilateral white matter with occipital accentuation, T2 high signal changes of the bilateral dorsal columns, extending from C1 to C5, as well as a right temporo-occipital periventricular cyst of 5 mm diameter. At the age of 2 years and 4 months, supratentorial white matter changes and spinal findings were still present. Moreover, long echo time MR spectroscopy from temporo-parietal white matter showed increased myo-inositol levels (SE 135 ms, 8 ml single voxel study). At the age of 3 years and 9 months, supratentorial T2 high signal white matter changes had increased markedly, as had the number of white matter cysts and the extent of spinal T2 high signal changes, which were now located more centrally. The increase of inositol levels on MR spectroscopy persisted. No gadolinium contrast enhancement was recorded (Fig. 1). A nerve conduction study at the age of 4 years and 11 months revealed no sign for peripheral neuropathy (motoric nerve conduction velocity of the ulnar nerve 52.0 m/s).

## Discussion

### The case report: differential diagnosis and comparison with the literature

Leukoencephalopathies with cysts encompass a heterogeneous group of disorders, and the differential diagnosis includes classical myelin diseases such as vanishing white matter disease and hypomyelinating leukodystrophies, the astrocytopathy Alexander disease, or inborn errors of metabolism such as mitochondrial disorders of the Leigh spectrum, lysosomal storage disorders such as Krabbe disease, and organic acidemias [48–51]. This case report and literature review highlights the importance of toxic–metabolic conditions in the differential diagnosis of leukoencephalopathy and adds another specific etiology to its spectrum.

The patient reported here shares many characteristics with previously published patients with B-HMBS-V (Additional file 1, Table 1), including delayed neuromotor development followed by neurodegeneration, characterized by motor decline and progressive spastic paraparesis during early life, as well as cystic white matter degeneration on neuroimaging. Whereas not showing a cataract, a pale optic disc was detected, which may mirror white matter disease. In line with previous reports, no porphyric attacks or response to porphyric stimuli were recorded thus far.

In the future, neurofilament light chain may be investigated as a potential biomarker in patients with B-HMBS-V, as it is already established in AIP [52].

### The correlation between HMBS variant and clinical severity

Thus far, seven different mutations, all missense mutations, have been described among patients with B-HMBS-V (T35M, L81P, A84D, R167Q, R167W, R173Q, and R225Q). The patient reported here proved compound heterozygous for the R167W and R167Q variants, respectively.

The R167W variant is a common cause for moderate AIP in heterozygous individuals [53, 54]. *In vitro*, the R167W HMBS enzyme shows an inefficient elongation process, leading to the accumulation of enzyme-intermediate complexes [55–57]. Another study also indicated a reduced stability of R167W, compared with R167Q, R173Q, and R173W [17]. However, homozygous R167W and R167Q HMBS enzymes showed higher *in vitro* activity (25% of normal) at the more physiological pH of 7.0, than at customary assay conditions with a pH of 8.0 (2% of normal), so that some *in vitro* results may not mirror the situation *in vivo* [58]. In AIP, the R173Q mutation may lead to an even more severe phenotype with more frequent porphyric attacks than R167W and R225Q [53]. The only reported patient with B-HMBS-V and the R173Q variant (R173Q/R167Q genotype) died at the age of 8 years [23]. Homozygosity for the R173Q variant even caused embryonic death in a murine model, and compound heterozygous R173Q/R167Q mice showed a very severe phenotype [35].

An approach to estimate the pathogenicity of HMBS variants *in silico* ranked the R225Q variant as benign, while R167W and R167Q were ranked pathogenic [12], but cases of heterozygous AIP with mild severity, caused by R225Q, prove the clinical significance of the variants [59, 60].

There are not many reports of the HMBS variant T35M, but clinical cases and *in vitro* data suggest a moderate pathogenicity [13, 61].

The variants A84D and L81P, which were found in homozygous state in two Lebanese siblings and one boy of Turkish ancestry, respectively, have not been described in the context of AIP before, but reduced HMBS activity in affected individuals with B-HMBS-V underlines their pathogenic impact [18, 25].

The reported cases with B-HMBS-V were diagnosed under various circumstances and in a very limited number, thus comparisons between them should be interpreted carefully. However, variants with low pathogenicity for heterozygous mutation carriers seem to cause a rather mild phenotype in B-HMBS-V and variants with medium pathogenicity for AIP can be linked to a more severe phenotype in B-HMBS-V. The described severity of the B-HMBS-V phenotype correlates with mortality in childhood, but not with the HMBS activity (Table 1).

**Table 1:** Phenotype shift

HMBS-variant	Phenotype AIP^†^ (heterozygous)	Phenotype B-HMBS-V^†,‡^	Mortality in childhood^‡^	HMBS activity *in vivo*^‡^ (% of norm)	References
A84D	No disease reported	+ (*n* = 2, both homozygous)	0/2	13–18	[18]
L81P	No disease reported	++ (*n* = 1, homozygous)	0/1	3	[25]
R225Q	+	+ (*n* = 4)	0/4	55–67	[18, 21, 60, 61]
R167Q	+	++ (*n* = 8)	1/8	14–67 (median 17)	[12, 18–24, 35, 54] present case
T35M	++	+++ (*n* = 1)	0/1	17	[13, 20, 61]
R167W	++	+++ (*n* = 5, 2 homozygous)	2/5 (both homozygous)	1–17 (median 15)	[17, 19, 53, 54] present case
R173Q	+++	++++ (*n* = 1)	1/1	ND	[22–24, 35, 54]
R173W	++++	Embryonic death?	ND	ND	[35, 53, 54]

The case reports of B-HMBS-V do not contain alleles, which are null allele mutations or are reported to cause a severe phenotype in AIP such as R173W [53]. Indeed, on the basis of the frequencies of pathogenic alleles [11, 12], one would expect about 0.3–0.4 cases per million of births. On the basis of the low number of reported cases in the medical literature thus far, it is therefore likely that HMBS variants with a lack of or a very low residual enzymatic activity cause embryonic death in humans with B-HMBS-V, such as the homozygous R173Q variant did in a murine model [35].

### The pathophysiology of B-HMBS-V

In acute hepatic porphyrias, neuronal heme deficiency, toxic effects of ALA, of PBG, or other abnormally elevated metabolites, as well as a depletion of substrates or cofactors as a result from disturbed heme synthesis [1], have been discussed to cause neurological disease, which includes T2 signal intense white matter lesions [8, 62–64]. Interestingly, when a patient with no inherited AIP received a liver transplant from a patient with AIP, he developed porphyria-typical biochemical signs and neurovisceral symptoms [65]. Liver transplantation, in turn, cures AIP in affected patients [66, 67]. Therefore, similar to the neurological involvement in AIP, neurodegeneration in B-HMBS-V could also be related to the liver defect. However, in wild-type mice, ALA does not cross the blood–brain barrier in general, but only in fenestrated capillaries of the circumventricular organs and tanycytes of the media eminence [33]. Moreover, an increase of ALA and PBG levels in the central nervous system (CNS) of mice with B-HMBS-V was attributed to intrathecal ALA and PBG synthesis, as no significant entrance of ALA or PBG into the CNS could be detected [35]. Hence, in B-HMBS-V, the neurological disease may originate from metabolic deficiency in the CNS itself.

Regarding acute encephalopathy in AIP, the current hypothesis from Pischik *et al*. postulates that it is caused by acute endothelial dysfunction resulting from the combination of abrupt hypertension, the syndrome of inappropriate antidiuretic hormone secretion (SIADH), and acute metabolic and inflammatory factors of hepatic origin [68]. Due to the lack of data about B-HMBS-V, it is difficult to assess the importance of endothelial dysfunction in its pathophysiology with certainty. It may be speculated, however, that it differs from its role in AIP, because neurological decline in B-HMBS-V does not appear to occur suddenly in response to porphyric stimuli but rather continuously, therefore acute endothelial dysfunction seems unlikely.

In CSF, although no reference values were available, we observed a high PBG level whereas the ALA level was just slightly elevated, respectively, compared with normal values in serum, so that toxic effects of PBG might be involved. However, literature data on the topic are limited. The distribution of PBG and ALA levels might result from intracerebral transformation of ALA to PBG by ALA dehydratase (ALAD) [17]. In a patient with AIP, considerably lower ALA and PBG levels in CSF compared with serum were observed, hence serum levels do not mirror CSF levels [34]. In a murine model Yasuda *et al*. showed that the elevation of PBG in CSF might not correlate with the situation in the brain tissue. Whereas CSF of mice with B-HMBS-V showed elevated PBG (74-fold) and elevated ALA (2.1-fold) levels, in brain tissue PBG was massively increased by several 100-fold and ALA was elevated 5.4-fold, compared with wild type mice [35]. This indicates that in B-HMBS-V, PBG accumulates in the CNS to a larger extent than ALA.

As for the toxic effects of ALA or PBG, neurotoxicity of ALA was indicated in several studies [30, 69]. Already, 0.01 μmol/l of extracellular ALA were shown to affect sodium channel activity in isolated rat hippocampal CA1 neurons [70]. Further, there is little evidence for neurotoxic effects of PBG, but when ALA or PBG were injected in a murine brain, they showed equal potential to cause seizures [71]. Simultaneously, clinical data show that a massive increase of ALA alone does not lead to cystic leukoencephalopathy. Patients with Doss porphyria, caused by biallelic pathogenic ALAD variants, show high elevations of ALA and coproporphyrin but not of PBG and uroporphyrin. They often exhibit severe peripheral polyneuropathy, but they do not develop an affection of the CNS. This implies that white matter disease is potentially elicited by the elevation of PBG [72–76].

By now, deficiency of neuronal heme does not seem to be the leading cause for leukoencephalopathy in B-HMBS-V. Surprisingly, in a murine model, the heme contents of brain and liver were even slightly higher in mice with homozygous HMBS variants than in mice with heterozygous HMBS variants [35].

Whereas a large spectrum of metabolites was elevated in the urine of the present patient, coproporphyrin was not elevated, an observation noted in other patients with B-HMBS-V [17, 19]. In contrast, a secondary elevation of coproporphyrin is common in AIP [77].

### Mitochondrial dysfunction in B-HMBS-V

Further reports discuss secondary mitochondrial dysfunction in patients with B-HMBS-V. Dixon *et al*. proved a disturbed mitochondrial function in peripheral blood mononuclear cells in patients with AIP and one severely affected child with B-HMBS-V and a lactate peak on MR-spectroscopy of white matter [20]. In these patients, the severity of mitochondrial dysfunction correlated with a more severe clinical phenotype. In two mouse models with B-HMBS-V, hypomyelination and compromised mitochondrial function in muscle and brain were reported [36, 37]. Solis *et al*. described a child with a HMBS activity below 1% and elevated serum lactate, serum pyruvate, and CSF lactate levels, respectively, although mitochondrial respiratory chain enzymes from muscle tissue proved normal [17]. Two patients, reported by Kevelam *et al*., also showed an increase in CSF lactate [21]. In contrast, in a neonatal patient with severe HMBS deficiency, Hessels *et al*. found normal values for blood lactate, urine organic acids, and serum acylcarnitines [25], and Pinder *et al*. reported on normal CSF lactate and urinary organic acids in a further severely affected child with homozygous VP [43]. In the patient reported here, elevated CSF lactate levels were found in one of two samples studied, whereas urine organic acids and serum carnitine levels proved normal. Brain MR-spectroscopy did not identify a lactate peak, but rather, a myoinositol peak, which indicates increased glial cell turnover, for example, in the case of damaged myelin sheets [78, 79].

### Treatment options for B-HMBS-V

Until now, there has been no evidence-based therapy for B-HMBS-V-associated CNS disease. A child with B-HMBS-V received heme infusions over five consecutive days, resulting in a significant decrease of urine ALA, PBG, and total porphyrins [20]. This shows that in B-HMBS-V, the regulatory mechanisms of heme synthesis are responsive to the substitution of heme. An adult woman with B-HMBS-V, who suffered from dysarthria, ataxia, and acute porphyric attacks, received a liver transplant. While her neurological condition improved during the subsequent year, it worsened thereafter despite no signs of transplant rejection [18]. A boy with Doss porphyria with severe polyneuropathy, normal cognition, and porphyric attacks received a liver transplant at the age of 6 years. No neurological improvement was recorded, however, he seemed to withstand porphyric challenges slightly better [80]. The efficiency of the siRNA givosiran, which silences ALAS1 mRNA and thus reduces the accumulation of the porphyrin precursors ALA and PBG [81], is unknown in patients with B-HMBS-V. In healthy individuals or patients with AIP, ALAS1 is upregulated to induce heme synthesis as a response to porphyric stimuli [82]. However, most patients with B-HMBS-V clinically do not respond to porphyric stimuli. In contrast to mice with heterozygous HMBS deficiency, mice with B-HMBS-V also do not respond to porphyric stimuli clinically or by an upregulation of ALAS1 mRNA. Therefore, the downregulation of ALAS1 by means of siRNA might not be effective [35]. Furthermore, givosiran is only effective for hepatic ALAS1 and not for the CNS metabolism [83]. Hence, its chance to improve the clinical course was considered unlikely, as thus far neither heme infusions nor the correction of the porphyria-related enzyme defect in the liver by transplantation had been effective in similar biallelic defects related to acute porphyrias [18, 20, 80]. With CRISPR/Cas9 techniques, it was already possible to correct a compound heterozygous porphyria with deficiency of uroporphyrinogen III synthase *in vitro* [84]. In the future, this therapy could gain more relevance *in vivo*.

For our patient, experimental treatments were not initiated after discussion with the parents.

## Conclusion

B-HMBS-V is a rare entity of cystic leukoencephalopathy with various clinical severity. Until now, little is known about the disease, and the symptoms and pathophysiology are most likely different to AIP. The nervous tissue damage in B-HMBS-V might be caused by the accumulation of PBG and other heme metabolites in the CNS. To date, there are no promising therapeutic approaches.

## Supplementary Information

Additional file 1. Overview of cases with B-HMBS-V, there is a list of reported cases of B-HMBS-V with their respective characteristics

## Data Availability

The dataset supporting the conclusions of this article is included within the article and its additional file.

## Abbreviations

AIP: Acute intermittent porphyria
ALA: 5-Aminolevulinic acid
ALAD: 5-Aminolevulinic acid dehydratase
ALAS1: 5-Aminolevulinic acid synthase 1
B-HMBS-V: Biallelic pathogenic hydroxymethylbilane synthase variants
CNS: Central nervous system
CSF: Cerebrospinal fluid
DNA: Deoxyribonucleic acid
GABA: Gamma-aminobutyric acid
HCC: Hepatocellular carcinoma
HMBS: Hydroxymethylbilane synthase
HPLC: High performance liquid chromatography
MRI: Magnetic resonance imaging
PBG: Porphobilinogen
siRNA: Small interfering RNA
VP: Variegate porphyria
