# Supplementary material for: Biallelic pathogenic hydroxymethylbilane synthase gene variants of a neurodegenerative disorder with progressive cystic leukoencephalopathy: a case report
Source: J Med Case Rep. 2026 Feb 23;20:121. doi: 10.1186/s13256-026-05879-2 (PMC12952037; doi:10.1186/s13256-026-05879-2)
Supplement: Supplementary file 1 — Additional file 1. Overview of cases with B-HMBS-V, there is a list of reported cases of B-HMBS-V with their respective characteristics [file 13256_2026_5879_MOESM1_ESM.pdf]

Additional file 1. Table 1. Overview of cases with B-*HMBS*-V

	Gen-der	Age at onset	Age at diagnosis	Age at last follow-up	genetics	Ancestry	Motor function impaired	Mentally impaired	White matter cysts	Myeli-no-pathy	Cata ract	Optic nerve disease	Seizures	Peripheral neuropathy	HMBS activity	Further abnormality
Present patient	m	12 mo	34 mo	4,7 y	R167Q c500G>A<br>R167W c499C>T	German	+++	++	+	+	-	-	+	-	16%	
Solis 2004	m	3 mo	13 mo	40 mo (died)	R167W c499C>T<br>R167W c499C>T	Spanish	+++	+++	+	+	+	-	-	+	1%	Hepatosplen omegaly
Solis 2004	m	2 mo	Diagnosis assumed	27 mo (died)	R167W c499C>T<br>R167W c499C>T<br>Assumed pathogenic	Spanish	+++	+++	ND	ND	ND	ND	ND	ND	ND	
Llewellyn 1992	f	18 mo	3 y	3 y	R167Q c500G>A<br>R167W c499C>T	British	++	ND	ND	ND	+	Right Hypo-plasia	+	ND	14%	Partial agenesis of cerebellar
Llewellyn 1992	m	no clinical disease	neonatal	18 mo	R167Q c500G>A<br>R167W c499C>T	British	-	-	ND	ND	-	-	-	ND	17%	
Beukeveld 1990	f	6 mo	Post-mortem	8 y (died)	R167Q c500G>A<br>R173Q c518G>A	Dutch	+++	+++	+	ND	ND	ND	-	ND	ND	
Dixon 2019	m	4-6 weeks	11 mo	13 mo	R167Q c500G>A<br>T35M c104C>T<br>Assumed base	Caucasian	+++	+++	ND	-	ND	ND	+	Clinical signs	17%	Lactate peak in MRI, heme therapy
Hessels 2004	m	9 mo	7 y	7 y	L81P c242T>C<br>L81P c242T>C	Turkish	++	++	ND	ND	ND	ND	-	ND	2-3%	Hepatosple-nomegaly, yellow teeth
Kevelam 2016	m	During teens	Adulthood	58 y	R167Q c500G>A<br>R225Q c674G>A	Dutch	+++	-	-	+	-	-	-	+	55%	
Kevelam 2016	f	4 y	Adulthood	63 y	R167Q c500G>A<br>R225Q c674G>A	Dutch	+++	+	-	+	+	Atrophy blind	-	+	67%	Cerebellar atrophy
Kevelam 2016	m	4 y	Adulthood	57 y	R167Q c500G>A<br>R225Q c674G>A	Dutch	+++	+	-	+	-	Atrophy	-	+	ND	Cerebellar atrophy
Stutterd 2021	f	7 y	36 y	43 y	A84D c251C>A<br>A84D c251C>A	Lebanese	++	-	-	+	+	-	-	+	18%	Acute porphyric attacks, Liver transplant
Stutterd 2021	m	Late child-hood	53 y	54 y	A84D c251C>A<br>A84D c251C>A	Lebanese	++	+	-	+	+	-	-	+	13%	
Stutterd 2021	m	13 y	40 y	45 y	R167Q c500G>A<br>R225Q c674G>A	Dutch	++	-	+	+	-	-	-	+	ND	
